# Characteristics of health-related quality of life and related factors in patients with brain tumors treated with rehabilitation therapy

**DOI:** 10.1186/s41687-022-00499-y

**Published:** 2022-09-06

**Authors:** Takahiro Watanabe, Shinichi Noto, Manabu Natsumeda, Shinji Kimura, Satoshi Tabata, Fumie Ikarashi, Mayuko Takano, Yoshihiro Tsukamoto, Makoto Oishi

**Affiliations:** 1 Rehabilitation Center, Niigata University Medical and Dental General Hospital, 754 Ichibancho, Asahimachidori, Chuo-ku, Niigata-shi, Niigata, 951-8510 Japan; 2 Major in Rehabilitation Sciences, Niigata University of Health and Welfare Graduate School, 1398 Shimami, Kita-ku, Niigata, Niigata Japan; 3 Department of Rehabilitation, Niigata University of Health and Welfare, 1398 Shimami, Kita-ku, Niigata, Niigata Japan; 4 Department of Neurosurgery, Brain Research Institute, Niigata University, 757 Ichibancho, Asahimachidori, Chuo-ku, Niigata-shi, Niigata, Japan

**Keywords:** Brain tumor, Quality of life, Activities of daily living, Rehabilitation

## Abstract

**Background:**

Rehabilitation therapy during hospitalization is effective in improving activities of daily living (ADL) and physical function in patients with brain tumors. However, there are few studies on the effect of rehabilitation therapy on health-related quality of life (HRQOL) in patients with brain tumors. Additionally, the EuroQol-5Dimension-5Level (EQ-5D-5L) index score has not been reported as an outcome. This study aimed to investigate the HRQOL of patients with brain tumors who underwent rehabilitation therapy and investigated the factors affecting the EQ-5D-5L index score from various perspectives, including various brain tumor type and recurrence. In addition, we examined the relationship between the EQ-5D-5L index score, disease-specific HRQOL scale, and ADL.

**Methods:**

Patients with brain tumors who underwent treatment and rehabilitation at Single tertiary care academic medical center were included in this cross-sectional study. We used the EQ-5D-5L, European Organisation for Research and Treatment of Cancer (EORTC) quality of life questionnaire core 30, and EORTC quality of life questionnaire brain cancer module to evaluate HRQOL. ADL were assessed using the functional independence measure (FIM). The relationship between each HRQOL assessment score and the FIM was analyzed, and the influence of related factors was assessed by multiple regression analysis.

**Results:**

This study included 76 patients. The EQ-5D-5L index score was 0.689 for all patients with brain tumors and 0.574 for those with glioblastomas, which was the lowest value. There was a moderate correlation between the EQ-5D-5L index score and FIM (r = 0.627, p < 0.001). In addition, the EQ-5D-5L index score was significantly correlated with most of the items of the disease-specific HRQOL scale. Multiple regression analysis revealed that glioblastoma histology (coefficient: − 0.373, p = 0.005) and recurrence (coefficient: − 0.273, p = 0.020) were independent factors affecting the EQ-5D-5L index score.

**Conclusions:**

Patients with glioblastoma undergoing rehabilitation have reduced HRQOL, which was influenced by glioblastoma histology and recurrence.

##  Introduction

Brain tumors are broadly classified as primary and metastatic brain tumors (MBTs), the latter of which are most commonly caused by the metastasis of lung cancer or breast cancer. Primary brain tumors affect approximately 7 people per 100,000 population worldwide every year, and the incidence is on the rise [1]. The treatment of brain tumors varies depending on the tumor category, generally consisting of multidisciplinary treatment with surgery, radiation therapy, and chemotherapy [2].

There has been great progress in the treatment methods for brain tumors in recent years, which have prolonged the survival of patients with brain tumors. Nevertheless, in some malignant brain tumors, the prognosis remains poor even with the aforementioned treatments. In particular, glioblastoma recurs at 6 months to 1 year on average. The reported median overall survival (OS) in glioblastoma is > 1.5 years, but the 5-years survival rate is still only 15% [2]. Similarly, the median OS for MBTs is 12.0 months, even for prostate cancer, which is considered to have the longest OS [3]. In patients with difficult-to-cure brain tumors and poor prognoses, it is important to improve and maintain health-related quality of life (HRQOL), which is a patient-reported outcome, as well as OS and progression-free survival.

The European Organisation for Research and Treatment of Cancer (EORTC) quality of life questionnaire core 30 (QLQ-C30) and EORTC quality of life questionnaire brain cancer module (BN20) are frequently used in the assessment of HRQOL in patients with brain tumors [4, 5]. These HRQOL scales are intended to capture disease-specific psychosomatic functions and symptoms. In recent years, the need for the economic evaluation of medical treatments has been increasing worldwide, generating more interest in utility scales. The EuroQol-5Dimension-5Level (EQ-5D-5L) is one of the most popular scales for calculating the utility index [6], but only a few studies have employed this scale in the evaluation of patients with brain tumors [7–9].

Rehabilitation therapy during hospitalization is reportedly effective in improving activities of daily living (ADL) and physical function in patients with brain tumors. Studies comparing patients with brain tumors to those with stroke and cerebral infarction with similar symptoms found comparable improvements in physical function, ADL, and home discharge rates [10–13]. In addition, even in patients with glioblastomas and MBTs, who are considered to have poor prognoses, a significant improvement in their total functional independence measure (FIM) score has been reported after rehabilitation [14, 15].

In contrast, there are few previous studies on the effect of rehabilitation therapy on HRQOL in patients with brain tumors [16]. Furthermore, the efficacy of rehabilitation therapy on HRQOL has not been examined in detail [17–20]. Additionally, the EQ-5D-5L index score has not been reported as an outcome. Thus, the efficacy of rehabilitation therapy differs between patients with brain tumors and those with stroke in terms of ADL and HRQOL, but the relationship between the two has not been fully investigated. In addition, few previous studies have analyzed the impact of disease factors such as brain tumor type and recurrence on HRQOL before and after rehabilitation therapy.

Clarifying the characteristics of HRQOL, the relationship between HRQOL and ADL, and the factors that affect HRQOL in patients with brain tumors at the time of hospital discharge will provide useful information for implementing rehabilitation therapy. Further, investigating the EQ-5D-5L index score of patients with brain tumors may provide evidence for the cost-effectiveness of rehabilitation treatment in the future. In the present report, we investigated the effects of brain tumor type and recurrence on the EQ-5D-5L index score. In addition, this study aimed to clarify the characteristics of HRQOL in different brain tumor types and its relationship with ADL.

## Methods

### Study design

This study uses a single-center cross-sectional study design. The design followed the international recommendations for strengthening the Reporting of Observational Studies in Epidemiology [21]. The study protocol was submitted and approved by the local ethical review committee (Approval No.:  2020-0380). The authors obtained written informed consent from patients who were hospitalized between April and September 2021. Furthermore, all data used in this study, including HRQOL and FIM, had been measured as part of routine care for patients with brain tumors since April 2016 at the institution. Therefore, patients undergoing rehabilitation from April 2016 to March 2019 we offered the opportunity to opt-out to use data.

### Patients

The participants comprised patients aged ≥ 20 years who were admitted to a single tertiary care university hospital for the treatment of brain tumors between April 2016 and September 2021. The patients were also undergoing physical and occupational therapy, or physical and occupational therapy with speech therapy. The exclusion criteria were based on previous studies [19, 22] and included: those who scored < 23 on the Mini Mental State Examination, those who had difficulty answering the HRQOL questions due to aphasia or severe higher brain dysfunction, and those who had difficulty answering the questions due to poor general health.

### Assessment of general health and HRQOL

The FIM was used to assess ADL, and the Karnofsky performance status (KPS) was used to assess general health. HRQOL was assessed using the QLQ-C30, BN20, and EQ-5D-5L. All parameters were assessed at the time of discharge. FIM and KPS were assessed by the therapist in charge, whereas in principle, patients were required to answer the HRQOL questions by themselves. The therapist in charge of the patient was allowed to assist the patient in answering the questions if the patient had certain limitations that impaired them from filling out the form. These limitations included reading impairment due to motor paralysis or visual field impairment caused by central nervous system disorders. Age, sex, brain tumor type, tumor location (right, left, other), surgery, radiotherapy, chemotherapy, first occurrence or recurrence, and destination were extracted from the medical records. Brain tumors were classified based on the results of pathological examination according to the WHO 2016 Pathological Classification of Brain Tumors [23]. Finally, based on previous studies [15, 22], the patients were classified into five groups: glioblastoma, grade III brain tumors (WHO grade III), primary central nervous system lymphomas (PCNSLs), MBTs, and grade I brain tumors (WHO grade I). The criteria used to determine whether surgery was performed were based on a previous study [24] and excluded biopsy from being considered a surgery. In patients with recurrent disease, history of previous surgery, radiotherapy, or chemotherapy was not included.

### Measurements

#### FIM

The FIM is an assessment of ADL, consisting of a motor category for self-care tasks (eating, grooming, bathing, dressing, toileting), sphincter control tasks (bladder management, bowel management), transfer tasks (bed-to-chair transfer, toilet transfer, tub or shower transfer), and locomotion tasks (walk or wheelchair, stairs), and a cognitive category for communication tasks (comprehension, expression) and social cognition tasks (social interaction, problem solving, memory). Each task is scored on a scale of 1–7 according to the level of independence, with 1 representing complete assistance and 7 representing complete independence. The total score ranges from 18 to 126, with a higher score indicating a greater degree of independence.

#### QLQ-C30 and BN20

In this study, QLQ-C30 Japanese version (3rd edition) and BN20 were used for evaluation. These are the HRQOL questionnaires developed by the EORTC, which have been reported to be valid and reliable [4, 5]. The QLQ-C30 is a disease-specific HRQOL assessment scale for patients with cancer. The QLQ-C30 is a 30-item, self-reported questionnaire containing the following domains: physical functioning (5 items), role functioning (2 items), cognitive functioning (2 items), emotional functioning (4 items), social functioning (2 items), global health status (2 items), nausea and vomiting (2 items), fatigue (3 items), pain (2 items), and single items for dyspnea, insomnia, anorexia, constipation, diarrhea, and financial difficulties.

The BN20 is a disease-specific measure of brain tumor symptoms. It contains 4 multi-item scales (future uncertainly, visual disorder, motor dysfunction, communication deficit) and 7 single items asking about headache, seizure, drowsiness, hair loss, itchy skin, weakness of legs and loss of bladder control.

The use of the above rating scales was approved by the EORTC Quality of Life Group. The QLQ-C30 and BN20 subscales are scored from 0 to 100 according to the scoring manual. A higher score on the QLQ-C30 functional scale and general health indicates better health, while a lower score on the QLQ-C30 symptom scale and BN20 indicates fewer complaints or better health.

#### EQ-5D-5L

The EQ-5D-5L is a generic preference-based measure of HRQOL developed by the EuroQol Group. EQ-5D-5L consists of five dimensions related to mobility, self-care, common activities, pain/discomfort, and anxiety/depression. Patients answer each item on a scale of 1–5 (no problems, slight problems, moderate problems, severe problems, and extreme problems). Initially developed by the EuroQol Group in 1987, the EuroQol-5Dimension-3Level (EQ-5D-3L) index was a five-item, three-level instrument. However, its sensitivity was insufficient, and a ceiling effect was identified. As a result, the five-level EQ-5D-5L was released to overcome these shortcomings [25]. In Japan, the EQ-5D-5L conversion table was completed in 2015, and the EQ-5D-5L index score reflecting Japanese values can be calculated [6]. The EQ-5D-5L utility index ranges from − 0.025 to 1.00 (full health status).

### Statistical analysis

A one-way analysis of variance (ANOVA) with Tukey post hoc analysis was performed to compare FIM total score, KPS, and HRQOL among the glioblastoma, WHO grade III, PCNSL, MBT, and WHO grade I groups. An unpaired t-test was used to compare the total FIM scores, KPS, and HRQOL between the two groups of first or recurrent disease. Additionally, effect size (ES) was calculated using Cohen’s d statistic. Cohen’s d [26] was used to estimate magnitude of standardized differences in means between groups; values were interpreted according to Cohen’s published guidelines (d = 0.2, small effect; d = 0.5, medium effect; d = 0.8, large effect). Pearson's correlation analysis was used to investigate the relationship between the EQ-5D-5L index score, FIM, and disease-specific HQOL scale. In accordance with Guilford's Rule of Thumb [27], the criterion for the strength of correlation was set as follows: |r|= 0–0.2 as “almost no correlation”, 0.2–0.4 as "weak correlation”, 0.4–0.7 as "moderately correlated", and 0.7–1.0 as "strongly correlated". Finally, a multiple regression analysis was performed to investigate the factors affecting the EQ-5D-5L index score at the time of hospital discharge, with the EQ-5D-5L index score as the dependent variable and age, sex, brain tumor type, and newly diagnosed or recurrent disease as independent variables. In this study, the forced imputation method of analysis was used to visually compare all independent variables with one other. The independent variables were selected with reference to previous studies that used the HRQOL scale and FIM total score as independent variables [15, 28]. Categorical data were transformed into dummy variables, and WHO grade I was used as the reference category for brain tumor type. A p-value of < 0.05 was regarded as being statistically significant, and all reported p-values were two-tailed. All statistical procedures were conducted using SPSS for Windows version 24.

## Results

### Patient characteristics

The patient characteristics are summarized in Table 1. The mean age of the patients was 61.1 years, and 59% were male. In addition, 74% of all patients were newly diagnosed, and 79% were discharged to home. The number of patients in each group was 21 in the glioblastoma group (27.6%), 10 in the WHO grade III group (13.2%), 10 in the PCNSL group (13.2%), 9 in the MBT group (11.8%), and 26 in the WHO grade I group (34.2%). The KPS for all patients was 86.3 ± 13.5, with no significant differences between brain tumor classifications in ANOVA (p = 0.065). None of the 10 patients in the PCNSL group had undergone surgical resection, whereas all patients in the MBT and WHO grade I groups had undergone surgery. In addition, none of patients in the WHO Grade I group received radiotherapy or chemotherapy.

**Table 1:** Patient characteristics

	All patients	Glioblastoma	WHO grade III	PCNSL	MBT	WHO grade I
	n = 76	n = 21	n = 10	n = 10	n = 9	n = 26
Sex, n (%)						
Male	45 (59)	15 (71)	6 (60)	5 (50)	8 (89)	11 (42)
Female	31 (41)	6 (29)	4 (40)	5 (50)	1 (11)	15 (58)
Age, years, mean ± SD	61.1 ± 12.5	58.7 ± 10.8	55.6 ± 11.7	64.5 ± 10.3	67.0 ± 9.5	61.8 ± 15.1
Tumor histology, n			Anaplastic astrocytoma 4			Meningioma 15
			Anaplastic oligodendroglioma 2			Schwannoma 6
			Anaplastic pleomorphic xanthoastrocytoma 2			Pituitary adenoma 2
			Anaplastic ependymoma 1			Hemangioblastoma 2
			NOS 1			Craniopharyngioma 1
KPS, mean ± SD	86.3 ± 13.5	84.8 ± 15.7	77.0 ± 12.5	92.0 ± 10.3	90.0 ± 7.1	87.7 ± 13.4
Tumor location, n (%)						
Right	27 (35)	10 (48)	6 (60)	5 (50)	3 (33)	3 (12)
Left	21 (28)	9 (43)	3 (30)	2 (20)	3 (33)	4 (15)
Both/other	28 (37)	2 (9)	1 (10)	3 (30)	3 (33)	19 (73)
Treatment, n (%)						
Surgical resection	59 (78)	17 (81)	7 (70)	0 (0)	9 (100)	26 (100)
Radiation	34 (45)	15 (71)	7 (70)	4 (40)	8 (89)	0 (0)
Chemotherapy	38 (50)	20 (95)	9 (90)	8 (80)	1 (11)	0 (0)
Recurrence, n (%)						
Yes	20 (26)	8 (38)	5 (50)	3 (30)	0 (0)	4 (15)
No	56 (74)	13 (62)	5 (50)	7 (70)	9 (100)	22 (85)
Discharge disposition, n (%)						
Discharged home	60 (79)	16 (76)	7 (70)	9 (90)	7 (78)	21 (81)
Transfer to a different hospital	16 (21)	5 (24)	3 (30)	1 (10)	2 (22)	5 (19)

*SD* Standard deviation, *KPS* Karnofsky performance status, *NOS* Not otherwise specified, *PCNSL* Primary central nervous system lymphoma, *MBT* Metastatic brain tumor

### Comparison of assessment scores among brain tumor types

**Fig. 1:**
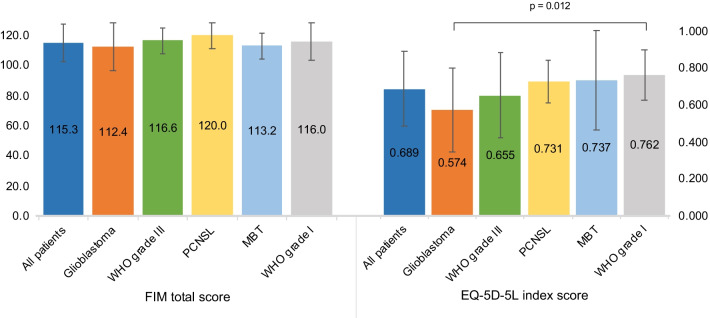
Comparison of FIM and EQ-5D-5L index score in tumor classification. PCNSL: Primary central nervous system lymphoma, MBT: Metastatic brain tumor

The assessment scores are summarized in Fig. 1 and Table 2. The FIM total score did not differ significantly among the brain tumor types (p = 0.438). In contrast, the EQ-5D-5L index scores showed significant differences by brain tumor classification (p = 0.048). Furthermore, by multiple comparisons, there was a significant difference between the glioblastoma group (0.574 ± 0.229) and the WHO Grade I group (0.762 ± 0.135) (p = 0.012), with a large ES of 1.03. In the disease-specific HRQOL scale, there were significant differences between groups in the items of emotional functioning (p = 0.015), financial difficulties (p = 0.002), and future uncertainty (p = 0.014). Multiple comparisons of these items showed significant differences between the glioblastoma group and WHO Grade I group, with each ES showing large values for emotional functioning (p = 0.001, ES = 1.14), financial difficulties (p < 0.001, ES = 1.45), and future uncertainty (p = 0.002, ES = 1.19).

**Table 2:** Comparison of disease-specific HQOL scale in tumor classification

	All patients	Glioblastoma	WHO grade III	PCNSL	MBT	WHO grade I	p value
	n = 76	n = 21	n = 10	n = 10	n = 9	n = 26	
	Mean ± SD	Mean ± SD	Mean ± SD	Mean ± SD	Mean ± SD	Mean ± SD	
*QLQ-C30 functional domains*^*a*^							
Physical functioning	67.5 ± 27.7	61.0 ± 31.6	61.3 ± 35.7	69.3 ± 25.8	70.4 ± 25.6	73.6 ± 23.5	0.612
Role functioning	56.4 ± 33.0	52.4 ± 28.5	53.3 ± 44.3	50.0 ± 31.4	64.8 ± 26.9	60.3 ± 35.3	0.75
Cognitive functioning	68.0 ± 25.2	63.5 ± 19.4	63.3 ± 35.8	61.7 ± 29.4	68.5 ± 26.9	75.6 ± 22.2	0.373
Emotional functioning	78.4 ± 16.1	68.7 ± 19.3	75.8 ± 12.1	82.5 ± 9.2	77.8 ± 20.4	85.9 ± 10.7	**0.015**
Social functioning	66.9 ± 29.4	57.1 ± 28.2	60.0 ± 28.5	76.7 ± 23.8	68.5 ± 30.6	73.1 ± 31.3	0.277
Global Health Status	52.3 ± 23.8	46.0 ± 24.2	48.3 ± 15.6	50.0 ± 27.5	57.4 ± 34.0	58.0 ± 20.5	0.422
*QLQ-C30 symptom domains*^*a*^							
Nausea and vomiting	5.7 ± 14.5	3.2 ± 6.7	18.3 ± 30.9	5.0 ± 11.2	5.6 ± 8.3	3.2 ± 10.6	0.605
Fatigue	40.3 ± 21.1	43.4 ± 12.6	46.7 ± 28.6	44.4 ± 18.1	39.5 ± 29.5	34.2 ± 21.3	0.448
Dyspnea	18.9 ± 27.9	19.0 ± 30.9	13.3 ± 23.3	26.7 ± 34.4	22.2 ± 23.6	16.7 ± 27.1	0.858
Pain	21.1 ± 21.3	30.2 ± 18.7	13.3 ± 17.2	18.3 ± 21.4	20.4 ± 33.1	17.9 ± 18.8	0.157
Insomnia	29.8 ± 29.1	33.3 ± 33.3	26.7 ± 34.4	36.7 ± 36.7	33.3 ± 16.7	24.4 ± 24.1	0.698
Appetite loss	21.1 ± 26.6	17.5 ± 22.7	20.0 ± 28.1	20.0 ± 23.3	40.7 ± 40.1	17.9 ± 23.5	0.631
Constipation	28.5 ± 29.2	33.3 ± 29.8	30.0 ± 33.1	30.0 ± 29.2	33.3 ± 33.3	21.8 ± 26.6	0.697
Diarrhea	10.1 ± 16.3	11.1 ± 16.1	3.3 ± 10.5	10.0 ± 16.1	22.2 ± 23.6	7.7 ± 14.3	0.253
Financial difficulties	33.3 ± 31.3	55.6 ± 28.5	26.7 ± 34.4	36.7 ± 33.1	29.6 ± 26.1	17.9 ± 23.5	**0.002**
*BN20 symptom domains*^*b*^							
Future uncertainty	34.3 ± 24.3	47.6 ± 24.0	36.7 ± 23.6	37.5 ± 26.7	32.4 ± 23.0	22.1 ± 19.1	**0.014**
Visual disorder	17.5 ± 28.3	10.1 ± 17.9	15.6 ± 32.8	15.6 ± 30.6	21.0 ± 34.0	23.9 ± 30.9	0.459
Motor dysfunction	23.4 ± 24.2	33.3 ± 27.2	28.9 ± 32.4	20.0 ± 30.5	17.3 ± 21.6	16.7 ± 11.9	0.164
Communication deficit	17.8 ± 22.4	22.8 ± 26.2	12.2 ± 15.2	10.0 ± 11.0	35.8 ± 31.3	12.8 ± 18.0	0.135
Headache	22.8 ± 23.9	19.0 ± 19.9	20.0 ± 23.3	16.7 ± 23.6	18.5 ± 17.6	30.8 ± 28.2	0.48
Seizure	1.3 ± 6.5	1.6 ± 7.3	6.7 ± 14.1	0.0 ± 0.0	0.0 ± 0.0	0.0 ± 0.0	0.068
Drowsiness	33.8 ± 28.5	36.5 ± 31.5	53.3 ± 28.1	26.7 ± 26.3	40.7 ± 36.4	24.4 ± 20.1	0.071
Hair loss	25.0 ± 30.9	34.9 ± 37.2	36.7 ± 33.1	23.3 ± 31.6	29.6 ± 30.9	11.5 ± 18.7	0.051
Itchy skin	18.0 ± 19.2	27.0 ± 22.7	20.0 ± 17.2	16.7 ± 17.6	18.5 ± 17.6	10.3 ± 15.7	0.107
Weakness of legs	40.8 ± 30.1	44.4 ± 26.5	46.7 ± 32.2	46.7 ± 32.2	44.4 ± 33.3	32.0 ± 30.5	0.558
Loss of bladder control	14.5 ± 23.9	14.3 ± 27.0	10.0 ± 16.1	13.3 ± 23.3	22.2 ± 23.6	14.1 ± 25.3	0.806

*SD* Standard deviation, *PCNSL* Primary central nervous system lymphoma, *MBT* Metastatic brain tumor

^a^In EORTC QLQ-C30, functional domains—higher scores are better; symptom domains—lower scores are better

^b^In EORTC BN20 symptom domains, lower scores are better

P value < 0.05 were written in boldface

### Comparison of recurrent and newly diagnosed groups

The results of the comparisons of recurrent and newly diagnosed groups are presented in Table 3. The KPS (p = 0.009, ES = 0.84), FIM total score (p = 0.048, ES = 0.63), EQ-5D-5L index score (p = 0.016, ES = 0.78), physical functioning score (p = 0.004, ES = 0.88), and role functioning score (p = 0.032, ES = 0.55) were significantly lower and the fatigue (p = 0.002, ES = 0.80), future uncertainty (p = 0.032, ES = 0.64), and drowsiness (p = 0.033, ES = 0.63) were significantly higher in the recurrence group than in the newly-diagnosed group. In addition, all items showed medium to large ES.

**Table 3:** Comparison of FIM, KPS, and HRQOL by recurrence and newly diagnosed groups

	Recurrence	Newly diagnosed	p value	ES (d)
	n = 20	n = 56		
	Mean ± SD	Mean ± SD		
FIM	109.8 ± 14.9	117.3 ± 10.6	**0.048**	0.63
KPS	78.5 ± 15.3	89.1 ± 11.6	**0.009**	0.84
EQ-5D-5L index score	0.577 ± 0.243	0.729 ± 0.175	**0.016**	0.78
*QLQ-C30 functional domains*^*a*^				
Physical functioning	50.7 ± 29.5	73.6 ± 24.6	**0.004**	0.88
Role functioning	43.3 ± 29.3	61.0 ± 33.2	**0.032**	0.55
Cognitive functioning	58.3 ± 25.6	71.4 ± 24.4	0.056	0.53
Emotional functioning	72.1 ± 20.1	80.7 ± 14.0	0.09	0.54
Social functioning	64.2 ± 31.2	67.9 ± 28.9	0.647	0.13
Global health status	48.3 ± 21.7	53.7 ± 24.6	0.364	0.23
*QLQ-C30 symptom domains*^*a*^				
Nausea and vomiting	12.5 ± 22.9	3.3 ± 9.2	0.094	0.66
Fatigue	52.2 ± 18.1	36.1 ± 20.7	0.002	0.8
Dyspnea	28.3 ± 34.7	15.5 ± 24.6	0.139	0.47
Pain	24.2 ± 19.8	19.9 ± 21.9	0.432	0.2
Insomnia	36.7 ± 37.3	27.4 ± 25.5	0.312	0.32
Appetite loss	23.3 ± 24.4	20.2 ± 27.5	0.641	0.12
Constipation	33.3 ± 32.4	26.8 ± 28.0	0.429	0.22
Diarrhea	5.0 ± 12.2	11.9 ± 17.3	0.06	0.43
Financial difficulties	38.3 ± 34.7	31.5 ± 30.1	0.443	0.22
*BN20 symptom domains*^*b*^				
Future uncertainty	45.4 ± 26.8	30.4 ± 22.2	**0.032**	0.64
Visual disorder	16.1 ± 23.5	18.1 ± 30.0	0.77	0.07
Motor dysfunction	30.6 ± 27.4	20.8 ± 22.6	0.166	0.41
Communication deficit	25.6 ± 25.5	15.1 ± 20.8	0.11	0.47
Headache	18.3 ± 22.9	24.4 ± 24.2	0.323	0.25
Seizure	5.0 ± 12.2	0.0 ± 0.0	0.083	0.81
Drowsiness	46.7 ± 31.3	29.2 ± 26.3	**0.033**	0.63
Hair loss	23.3 ± 37.6	25.6 ± 28.4	0.808	0.07
Itchy skin	16.7 ± 20.2	18.5 ± 19.0	0.733	0.09
Weakness of legs	41.7 ± 30.4	40.5 ± 30.3	0.881	0.04
Loss of bladder control	23.3 ± 30.8	11.3 ± 20.4	0.117	0.51

*FIM* Functional independence measure, *KPS* Karnofsky performance status, *SD* Standard deviation, *ES* Effect size

^a^In EORTC QLQ-C30, functional domains—higher scores are better; symptom domains—lower scores are better

^b^In EORTC BN20 symptom domains, lower scores are better

p value < 0.05 were written in boldface

### Correlations among the EQ-5D-5L index score, FIM, and disease-specific HRQOL scale

The correlations among the EQ-5D-5L index score, FIM, and disease-specific HRQOL scale are shown in Table 4. There was a moderate correlation between the EQ-5D-5L index score and FIM (r = 0.627, p < 0.001). Furthermore, the EQ-5D-5L index score and the disease-specific HRQOL scale showed significant correlations for all items with the exception of headache, hair loss, and itchy skin. In particular, strong correlations were observed with physical functioning (r = 0.723, p < 0.001). In contrast, only physical functioning (r = 0.610, p < 0.001) and dyspnea (r = − 0.433, p < 0.001) showed more than a moderate correlation between FIM and the disease-specific HRQOL measure.

**Table 4:** Correlation between EQ-5D-5L index score, FIM, and disease-specific HQOL scale

	FIM	EQ-5D-5L index score
	Pearson’s r	Pearson’s r
EQ-5D-5L index score	0.627**	
*QLQ-C30 functional domains*^*a*^		
Physical functioning	0.610**	0.723**
Role functioning	0.325**	0.602**
Cognitive functioning	0.354**	0.584**
Emotional functioning	0.213	0.651**
Social functioning	0.171	0.482**
Global health status	0.129	0.430**
*QLQ-C30 symptom domains*^*a*^		
Nausea and vomiting	− 0.098	− 0.228*
Fatigue	− 0.331**	− 0.669**
Dyspnea	− 0.433**	− 0.496**
Pain	− 0.281*	− 0.533**
Insomnia	− 0.184	− 0.400**
Appetite loss	− 0.139	− 0.412**
Constipation	− 0.110	− 0.274*
Diarrhea	− 0.157	− 0.249*
Financial difficulties	− 0.077	− 0.406**
*BN20 symptom domains*^*b*^		
Future uncertainty	− 0.174	− 0.566**
Visual disorder	− 0.142	− 0.256*
Motor dysfunction	− 0.095	− 0.448**
Communication deficit	− 0.145	− 0.374**
Headache	0.115	0.003
Seizure	0.073	− 0.231*
Drowsiness	− 0.180	− 0.524**
Hair loss	0.124	− 0.141
Itchy skin	0.081	− 0.030
Weakness of legs	0.017	− 0.273*
Loss of bladder control	− 0.265*	− 0.377**

*FIM* Functional independence measure

^a^In EORTC QLQ-C30, functional domains—higher scores are better; symptom domains—lower scores are better

^b^In EORTC BN20 symptom domains, lower scores are better

Pearson’s r ≧| 0.400 | were written in boldface. ∗∗ p < 0.01, *p < 0.05

### Multiple regression analysis for EQ-5D-5L index score

Multiple regression analysis was performed on the EQ-5D-5L index score, with age, sex, brain tumor type, surgery, radiotherapy, chemotherapy, and first occurrence or recurrence as independent variables (Table 5). Glioblastoma (standard partial regression coefficient: − 0.373, p = 0.005) and recurrence (standard partial regression coefficient: − 0.273, p = 0.020) were identified as factors affecting the EQ-5D-5L index score.

**Table 5:** Multiple regression analysis with EQ-5D-5L index score as the dependent variable

	B	β	95% Confidence interval		p value
			Lower	Upper	
Intercept	0.907		0.629	1.185	< 0.001**
Age	− 0.002	− 0.097	− 0.005	0.002	0.388
*Sex*					
Female (ref)					
Male	− 0.017	− 0.041	− 0.110	0.077	0.719
*Tumor histology*					
WHO grade I (ref)					
Glioblastoma	− 0.170	− 0.373	− 0.287	− 0.052	0.005**
WHO grade III	− 0.077	− 0.127	− 0.224	0.071	0.305
PCNSL	− 0.009	− 0.016	− 0.152	0.133	0.895
MBT	− 0.044	− 0.070	− 0.199	0.111	0.575
*Recurrence*					
No (ref)					
Yes	− 0.126	− 0.273	− 0.232	− 0.020	0.020*

B: Partial regression coefficient, β: Standardized regression coefficient, PCNSL: Primary central nervous system lymphoma, MBT: Metastatic brain tumor

Multiple R2: 0.220, Adjusted R2: 0.140. **p < 0.01, *p < 0.05

## Discussion

This study aimed to investigate the effects of brain tumor type and recurrence on the EQ-5D-5L index score and to clarify the characteristics of HRQOL in different brain tumor types and its relationship with ADL.

The mean EQ-5D-5L index score at the time of hospital discharge for all patients with brain tumors in this study was 0.689 ± 0.205 (mean age 61.1 years). The WHO grade I group had the highest score of 0.762 ± 0.135 (mean age 61.8 years) and the glioblastoma group had the lowest score of 0.574 ± 0.229 (mean age 58.7 years). In Japan, the EQ-5D-5L index score for patients with brain tumors has not been reported previously, and in other countries, Wagner et al. [7] reported a mean index score of 0.72 in 3-months postoperative patients with benign meningiomas. The EQ-5D-5L index score of the WHO grade I group in this study was comparable, although simple comparison is difficult because the EQ-5D-5L index score is calculated using a country-specific conversion table. However, the mean EQ-5D-5L index score of the general population in Japan was reported to be 0.936 in the 50 s and 0.911 in the 60 s [29]. In the case of patients with various types of outpatient cancers aside from brain tumors, the reported value was 0.827 [30]. In addition, the mean score was 0.52 (mean age 57 years) in stroke patients, who are expected to present with similar functional impairment [31]. The EQ-5D-5L index score of the brain tumor patients in this study was lower than that of the general population and patients with other cancers, although the results should be interpreted with caution regarding the different effects of the time of assessment, age, and disease. Furthermore, the values were similar between the current glioblastoma group and previous reports of stroke. In the present study, there were significant differences in emotional functioning, financial difficulties, and future uncertainty among brain tumor types. In addition, the glioblastoma group showed the lowest values for all scales. Budrukkar et al. [22] reported that the Global Health Status of the QLQ-C30 was significantly lower in the high-grade glioma (HGG) group than in the low-grade glioma (LGG) group. In a study of glioma patients treated with rehabilitation therapy during hospitalization, Umezaki et al. [28] found that the HGG group had fewer complaints of QLQ-C30 constipation and more complaints of BN-20 hair loss and itchy skin than did the LGG group. These previous studies and the current results differed in the items that showed significant differences. This may have been due to differences in the types of brain tumors targeted and the associated treatment regimens, as well as the number of hospitals treating brain tumor patients in the study area. In general, glioblastoma is the most malignant brain tumor and has a poor prognosis. Therefore, it may not be surprising that the lowest values EQ-5D-5L index score and worst scores on other scales/domains. However, it is interesting to note that in the present study, the scores of HRQOL items reflecting psychological aspects were lower in the glioblastoma group than in the other groups, even though there was no significant difference in FIM total score by brain tumor classification. Glioblastomas carry a poorer prognosis than other brain tumors, aside from MBTs, and may cause psychological problems. Indeed, patients with glioblastoma have been reported to have more depressive symptoms than patients with gastric, urological, breast, and lung cancers [32]. Further, most brain tumors classified as WHO grade I can be treated with surgery alone, but brain tumors classified as WHO grade II or higher often require radiation therapy or chemotherapy in addition to surgery. Moreover, these treatments may be continued after hospital discharge. These factors may be related to the emotional functioning, financial difficulties, and future uncertainty scores of the glioblastoma group. However, within the scope of this study, we have not been able to examine the above points, and they are only inferred.

A previous study in acute stroke patients reported a significant correlation between the EQ-5D-5L index score and FIM motor items [33] and Barthel index [34]. In contrast, a previous study of patients with brain tumors reported no correlation between the total FIM score at discharge and the Functional Assessment of Cancer Therapy-Brain (FACT-Br), a disease-specific HRQOL scale [18]. In the present study, we found a significant correlation between the FIM and EQ-5D-5L index score. However, in the FIM total score and disease-specific HRQOL scale, the items that showed significant correlations were limited to those related to physical function. This finding was similar to those of previous studies, although the HRQOL scale used was different. However, our results are noteworthy in that the EQ-5D-5L index score and the disease-specific HRQOL scale showed significant correlations for all items with the exception of headache, hair loss, and itchy skin on the BN20. Hirose et al. [30] reported a correlation between changes in adverse events and EQ-5D-5L index scores in patients with cancer. In addition, the EQ-5D-3L index score of patients with brain tumors is reportedly associated with the emotional well-being item of the FACT-Br [35] and anxiety and depression symptoms [36]. The correlations between EQ-5D-5L index scores and the QLQ-C30 and BN20 in this study were similar to those in previous studies, although the target diseases and HRQOL assessment scales were different. Coomans et al. [24] reported the impact of HRQOL on OS in patients with gliomas, but the added value was low, indicating the limitations of using HRQOL as a prognostic indicator of OS. However, Edelstein et al. [32] stated that the limitation of activity and participation due to glioblastoma is a factor that interferes with subjective well-being and mentioned the possibility of rehabilitation therapy to improve HRQOL. Similarly, in addition to training to improve ADL as indicated by the HRQOL assessment, the importance of rehabilitation treatment for patients with brain tumors, which is largely affected by individual complaints, is demonstrated in this study.

Furthermore, we investigated the influence of factors such as brain tumor type and recurrence on the EQ-5D-5L index score. The results of multiple regression analysis showed that glioblastoma and recurrence were the most influential factors. Vera et al. [37] investigated the effect of different brain tumor classifications on the EQ-5D-3L index score in patients with gliomas who were undergoing outpatient treatment. After dividing the patients into two groups, grade II/III and grade IV, we reported that the grade of the brain tumor was not a factor affecting the EQ-5D-3L index score. Similar results were also reported in a study of postoperative HGG and LGG patients [38]. In the present study, glioblastoma reduced the EQ-5D-5L index score, and this finding differed from those of previous studies. However, these previous studies were limited to the glioma population. In our study, we used the EQ-5D-5L index and further divided the brain tumor classifications into five groups, which we believe is a new finding.

There are several reports on the impact of brain tumor recurrence on HRQOL. Osada et al. [39] point out that HRQOL is reduced by recurrence because of the effect of tumor expansion as the disease progresses. Furthermore, Okita et al. [40] also showed that HRQOL in patients with Grade II glioma is maintained in the long term, but functional decline due to recurrence may reduce HRQOL. Similar observations have been reported for glioblastoma [41], PCNSL [42], and meningiomas [43]. On the other hand, some have reported that recurrence does not affect HRQOL or ADL [38, 44]. However, these studies had limited study populations and varied in the time from recurrence to HRQOL survey. The recurrence group in this study was a population hospitalized for treatment associated with recurrence and may have been more likely to reflect the impact of recurrence on HRQOL. Therefore, it is possible that the deterioration of function and exacerbation of symptoms associated with recurrence affected the EQ-5D-5L index score. Even in relapsed cases, HRQOL may be maintained or improved with the course of treatment [45, 46].

This study was a cross-sectional survey and did not examine pre- and post-treatment. Therefore, we recognize that longitudinal surveys and studies are needed to understand the impact of rehabilitation in brain tumor patients, and plan address this in future studies.

## Limitations

There are several limitations to this study. First, this study was conducted at a single institution and was limited to patients with brain tumors who underwent rehabilitation treatment. In addition, patients with poor general health, cognitive decline, or aphasia were excluded. Therefore, the results are not generalizable to all patients with brain tumors. In addition, because this was a cross-sectional study, we were not able to compare the findings before and after rehabilitation treatment, nor were we able to examine changes after hospital discharge.

Second, in the HRQOL survey of brain tumor patients, the influence of differences in analysis methods has been pointed out [47]. For example, in a single-item scale, even a slight difference between "not at all" and "a little" responses indicates a large change in scores. In addition, if HRQOL items that improve, maintain, or worsen differ among individual subjects, the results of group-level analysis may average the scores of patients who reported decline and those who reported improvement, obscuring the true characteristics. Therefore, the need for analysis at the individual patient level has been pointed out. The purpose of this study was to examine the characteristics of HRQOL among brain tumor classifications and its effect on the EQ-5D-5L index score. On the other hand, the study did not longitudinally examine individual patients, which is a limitation of this study and should be addressed in future studies.

Third, previous studies have reported that a history of epilepsy and impaired cognitive function [48], as well as tumor location and size [49], affect HRQOL, but we were unable to examine their effects in this study. In addition, the late effects of radiotherapy and chemotherapy may have affected HRQOL after the study. Since brain tumors are rare, there is a need to evaluate a greater number of cases by conducting multicenter studies. Furthermore, in the present study, the analysis was only performed at discharge after rehabilitation therapy. We are currently conducting continuous surveys before and after rehabilitation treatment and after discharge from the hospital in order to longitudinally understand the effect of rehabilitation on ADL and HRQOL.

## Conclusion

In this study, we investigated HRQOL in patients with brain tumors treated with rehabilitation therapy and examined factors affecting EQ-5D-5L index scores in terms of various types of brain tumors and recurrence. In addition, we examined the relationship between the EQ-5D-5L index score, disease-specific HRQOL scale, and FIM total score. The EQ-5D-5L index score of the patients in this study was lower than that of the general adult population. In addition, the glioblastoma group had the lowest EQ-5D-5L index score among all brain tumor types. In addition, the EQ-5D-5L index score was significantly correlated with most of the items of the disease-specific HRQOL scale in addition to the total FIM score. Multiple regression analysis revealed that glioblastoma and recurrence were factors that significantly influenced the EQ-5D-5L index score. The results of our study may provide useful information for the rehabilitation of patients with brain tumors.

## Data Availability

The datasets generated and/or analyzed during the current study are available from the corresponding author on reasonable request.

## Abbreviations

ADL: Activity of daily living
ANOVA: A one-way analysis of variance
BN20: Brain cancer module
ES: Effect size
EORTC: European Organisation for Research and Treatment of Cancer
EQ-5D-3L: EuroQol-5Dimension-3Level
EQ-5D-5L: EuroQol-5Dimension-5Level
FACT-Br: Functional Assessment of Cancer Therapy-Brain
FIM: Functional independence measure
HGG: High-grade glioma
HRQOL: Health-related quality of life
KPS: Karnofsky performance status
LGG: Low-grade glioma
MBT: Metastatic brain tumor
OS: Overall survival
PCNSL: Primary central nervous system lymphoma
QLQ-C30: Quality of life questionnaire core 30
